# Dynamic Changes in Circulating Osteogenic Progenitor Cells Following TAVI: Implications for Vascular Remodeling—EPC and EPC-OCN Dynamics After TAVI

**DOI:** 10.3390/jcm15072752

**Published:** 2026-04-05

**Authors:** Lia Schoenfeld, Pablo Codner, Merry Abitbol, Ben Cohen, Dorit Leshem Lev, Amos Levi, Ariel Nakache, Guy Witberg, Yeela Talmor Barkan, Ran Kornowski, Leor Perl

**Affiliations:** 1 Department of Cardiology, Rabin Medical Center, Beilinson Campus, Petah-Tikva 4941492, Israel; liasheinfeld@gmail.com (L.S.); merry.abitbol@gmail.com (M.A.); i916bc@gmail.com (B.C.); amos.levi@gmail.com (A.L.); ariel.nako@gmail.com (A.N.); vitberguy@gmail.com (G.W.); talmor.yeela@gmail.com (Y.T.B.); ran.kornowski@gmail.com (R.K.); leorperl@gmail.com (L.P.); 2 Gray Faculty of Medicine, Tel Aviv University, Tel Aviv 6997801, Israel; 3 Felsenstein Research Center, Rabin Medical Center, Petah-Tikva 4941492, Israel; doritme39@gmail.com

**Keywords:** aortic stenosis, endothelial progenitor cells, osteocalcin, transcatheter aortic valve implantation, vascular calcification

## Abstract

**Background**: The prevalence of severe aortic stenosis (AS) is increasing, in accordance with a longer life expectancy. Aortic valve calcification is a multifactorial pathological process involving a complex interplay between different types of regenerative cellular and genetic factors. Among these cells, endothelial progenitor cells (EPCs) and their osteoblastic phenotype subpopulation (EPC-OCNs) have been implicated in vascular remodeling and disease progression. **Objectives**: To assess longitudinal changes in EPC and EPC-OCN levels in patients with severe symptomatic AS undergoing transcatheter aortic valve implantation (TAVI). **Methods**: In this prospective observational study, 65 patients with severe AS undergoing TAVI were enrolled. Circulating EPC and EPC-OCN levels were quantified by flow cytometry before the procedure, at 4 ± 1 days, and at 90 ± 29 days after TAVI. EPCs were defined by expression of CD133, CD34, and VEGFR-2. **Results**: Circulating EPC levels remained unchanged throughout the follow-up. In contrast, circulating EPC-OCNs increased significantly over time. Specifically, CD133+/VEGFR-2+/OCN+ cells rose from 2.50% to 6.25%, CD34+/VEGFR-2+/OCN+ from 2.04% to 4.05%, and VEGFR-2+/OCN+ from 1.46% to 3.01% (all *p* < 0.01). This suggests an osteogenic response to TAVI, while classical endothelial repair mechanisms were not systemically activated. **Conclusions**: EPC-OCNs increased significantly following TAVI, possibly reflecting ongoing tissue remodeling or calcification processes. In contrast, the stability of classical EPCs levels suggests limited systemic endothelial regeneration. These observations underscore the potential role of EPC-OCNs as markers or modulators of pre- and post-TAVI vascular remodeling.

## 1. Introduction

Calcific aortic stenosis (AS) and degenerative mitral regurgitation are the most prevalent valvular diseases in developed countries [1]. Aortic valve calcification is an active, multifactorial pathological process that involves different types of cells and genes [2,3]. Histologically, the aortic valve is composed of valvular interstitial cells (VICs), endothelial cells, collagen, glycosaminoglycans and elastin [4,5]. Calcified nodules develop due to deleterious stimuli, and include osteoblast expression, cell proliferation, and atherosclerosis [6].

The number and function of endothelial progenitor cells (EPCs) correlate inversely with cardiovascular risk [7]. EPCs are positive for CD133/CD34/VEGFR-2 (KDR) cell surface markers early after entering the systemic circulation, losing the CD133 as they mature [8]. Patients with severe AS have lower levels of circulating EPCs (CD34+/VEGFR-2+/OCN+) than controls and EPC levels inversely correlate with AS severity [9,10]. Moreover, in patients with similar severity of AS, a lower number of EPCs was associated with increased rates of cardiovascular events at follow-up [11]. On the contrary, patients with severe AS have elevated numbers of total circulating EPCs with osteoblastic phenotype (EPC-OCNs) (CD133+/OCN+, CD34+/CD133+/OCN+, and CD133+/VEGFR-2+/OCN+) [12]. EPC-OCNs are unique cells that express both endothelial and osteoblastic markers, and their levels increase significantly as AS progresses. These cells are hypothesized to contribute to valve calcification by engrafting into the endothelial layer and activating osteoblastic VICs upon valve injury. In severe AS, EPC-OCNs may play a role in neovascularization and osteogenesis within the valve, processes supported by the increased abundance of EPC-OCNs in circulation and their detection in calcified aortic valve tissues. EPC-OCNs actively participate in the pathogenesis and progression of calcific AS [12].

Transcatheter aortic valve implantation (TAVI) provides a clinically relevant setting in which major hemodynamic and biological changes occur over a short time frame. Severe AS is associated with abnormal shear stress and inflammatory activation, and previous work has shown that relief of valvular obstruction by TAVI may attenuate shear stress-related inflammatory responses. At the same time, the procedure itself induces acute vascular and valvular injury and may trigger dynamic changes in circulating cellular populations. Therefore, TAVI offers a unique human model in which to examine the temporal balance between endothelial repair-associated and osteogenic progenitor cell responses after valve intervention [13,14].

Despite accumulating evidence linking EPC-OCNs with AS severity and valve calcification, little is known about their short- and intermediate-term behavior after TAVI. Characterizing the temporal response of circulating EPC and EPC-OCN subsets following valve implantation may improve our understanding of post-procedural vascular and valvular remodeling and help determine whether these cell populations have potential relevance as biomarkers of biological response after intervention [12,15].

We aimed to assess the changes in EPCs and EPC-OCNs levels at different time points in patients with severe symptomatic AS undergoing TAVI.

## 2. Methods

### 2.1. Study Design and Patient Population

This observational, non-blinded, non-interventional prospective study enrolled 65 patients with severe symptomatic AS undergoing TAVI. Levels of EPCs and EPC-OCNs were measured before and after valve implantation at two additional time points for assessment. Patients were excluded if they had experienced acute coronary syndrome or undergone interventional coronary angiography in the past three months, had a bicuspid aortic valve, active malignant disease, sepsis or acute infection, active inflammatory or rheumatic disease, cardiac or vascular surgery in the past six months, chronic liver failure, moderate to severe chronic kidney disease (eGFR < 60 mL/min/1.73 m^2^), early post-procedural complications (such as death, stroke, or major bleeding), significant anemia (hemoglobin < 10 mg/dL), leukopenia (<4 K/mL), thrombocytopenia (platelets < 130 K/mL), or were receiving anticoagulation therapy.

All patients had severe symptomatic AS and were scheduled for TAVI. Patients were assessed by a dedicated TAVI clinic, underwent transthoracic echocardiography, gated cardiac CT and other evaluations as required. The institutional Heart Team assigned treatments.

After written informed consent was obtained, three blood samples were collected from each patient before TAVI, at 4 ± 1 days after the procedure, and at 90 ± 29 days after TAVI. Demographic, procedural, and post-procedural data were recorded in a dedicated database. The study was approved by the Rabin Medical Center Helsinki Committee.

### 2.2. EPC Quantification

Peripheral blood mononuclear cells were isolated from EDTA-anticoagulated blood by Ficoll density-gradient centrifugation, as previously described [16]. Surface marker expression was quantified by flow cytometry. Briefly, 1 × 10^6^ cells per sample were incubated for 20 min at 4 °C in the dark with fluorochrome-conjugated monoclonal antibodies against VEGFR-2 (clone 89106, FITC; R&D Systems, Minneapolis, MN, USA), CD133 (clone AC133, PE; Miltenyi Biotec, Bergisch Gladbach, Germany), CD34 (clone AC136, PE; Miltenyi Biotec, Bergisch Gladbach, Germany), and osteocalcin (OCN; R&D Systems, Minneapolis, MN, USA). Appropriate isotype-matched control antibodies were included for each fluorochrome to assess nonspecific binding. After incubation, cells were washed and analyzed using a FACSCalibur flow cytometer (Becton Dickinson, Franklin Lakes, NJ, USA). At least 100,000 events were acquired per sample. Compensation was performed using single-stained controls.

A stepwise gating strategy was used to identify EPC and EPC-OCN populations. First, debris was excluded according to forward scatter (FSC) and side scatter (SSC) characteristics. Second, doublets were excluded by gating on FSC-A versus FSC-H to retain single cells only. Third, mononuclear cells were selected based on their FSC/SSC profile. Within the mononuclear cell population, EPCs were defined as cells expressing VEGFR-2 together with CD34 or CD133. EPC-OCN populations were defined as VEGFR-2-positive cells co-expressing osteocalcin. Early EPC-OCNs were identified as CD133+/VEGFR-2+/OCN+ cells, whereas more mature EPC-OCNs were identified as CD34+/VEGFR-2+/OCN+ cells. Fluorescence thresholds were determined using isotype controls, and the same gating strategy was applied consistently across all samples [17].

To enhance reproducibility, all samples were processed according to a standardized protocol and analyzed in duplicate. The same acquisition settings, compensation approach, and gating framework were applied uniformly across samples.

### 2.3. Statistical Analysis

Descriptive statistics were used to present continuous variables as means ± standard deviations and categorical variables as percentages. Paired *t*-tests compared EPC and EPC-OCN levels before and after TAVI for each subtype (CD133, CD34, VEGFR-2). ANOVA assessed changes across the three time points (pre-TAVI, post-TAVI, and late). A significance level of α = 0.05 was applied, with *p* < 0.05 considered statistically significant. Analyses were conducted with IBM SPSS version 28.0 (IBM Corp. Armonk, NY, USA).

## 3. Results

### 3.1. Patient Characteristics

A total of 65 patients with severe symptomatic AS were analyzed, the mean age was 79.0 ± 7.7 years, with 58.4% females. Mean body mass index was 28.4 ± 4.2 kg/m^2^. Common cardiovascular comorbidities included hypertension (73.8%), diabetes mellitus (46.1%), ischemic heart disease (35.3%), prior PCI (32.3%), and previous stroke or transient ischemic attack (12.3%). Atrial fibrillation and previous coronary artery bypass grafting were present in 7.6% of patients each, while peripheral vascular disease was documented in 10.7%.

Baseline echocardiographic and imaging parameters were consistent with advanced aortic stenosis, with a mean left ventricular ejection fraction of 57.3 ± 10.1%, mean aortic valve gradient of 50.6 ± 15.8 mmHg, maximal gradient of 81.9 ± 25.3 mmHg, and an effective aortic valve area of 0.6 ± 0.1 cm^2^. The mean aortic valve calcium score was 2536 ± 1484 AU. Mean hemoglobin level was 11.9 ± 1.4 g/dL and mean platelet count was 221.0 ± 59.8 × 10^3^/µL. Regarding baseline medical therapy, 71.9% of patients were treated with aspirin, 84.2% with a P2Y12 inhibitor, and 78.9% with statins.

### 3.2. Circulating EPC and EPC-OCN Levels

**Figure 1:**
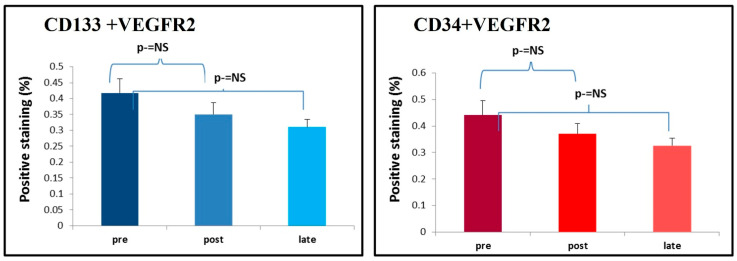
EPC levels over the follow-up time period. This figure shows the mean levels of EPC in PBMCs isolated from patients over three time points: pre-procedural, early post-procedural, and late post-procedural. EPCs levels remaining stable through the early and late follow-up (p-=NS).

No significant changes were observed in circulating EPC levels throughout the follow-up period. CD34+/VEGFR-2+ EPCs decreased slightly from 0.443% pre-procedurally to 0.371% post-TAVI and remained low at 0.378% during late follow-up, with no statistically significant difference. Similarly, CD133+/VEGFR-2+ EPCs showed a slight decline from 0.421% at baseline to 0.333% post-TAVI and 0.326% at late follow-up, with no statistically significant differences (Figure 1, Table 1).

In addition, the proportion of OCN+ cells lacking EPC markers remained stable over time, showing no significant change during follow-up.

**Figure 2:**
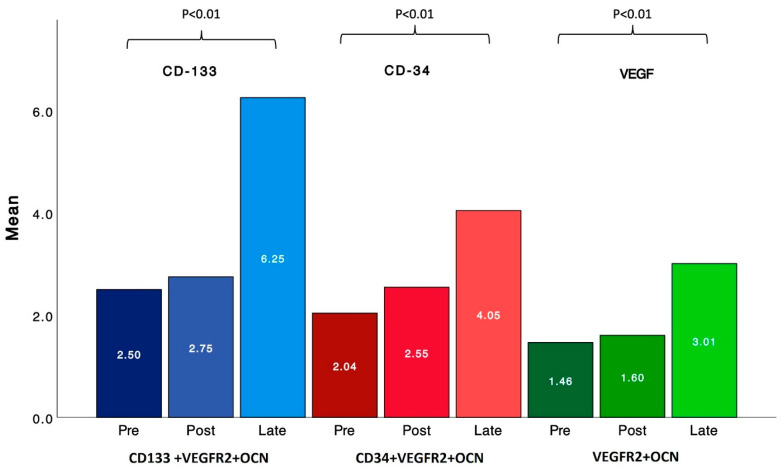
EPC-OCN levels Over the follow-up time period. This figure illustrates the mean levels of EPC-OCN levels as presented by the cell markers: CD133+VEGFR-2+OCN, CD34+VEGFR-2+OCN, and VEGFR-2+OCN, in patients over three time points: pre-procedural, early post-procedural, and late post-procedural. The significant increase in levels of this cell markers from baseline to the late post-procedural stage (*p* < 0.01) suggests an increase in EPC-OCN activity over time.

**Figure 3:**
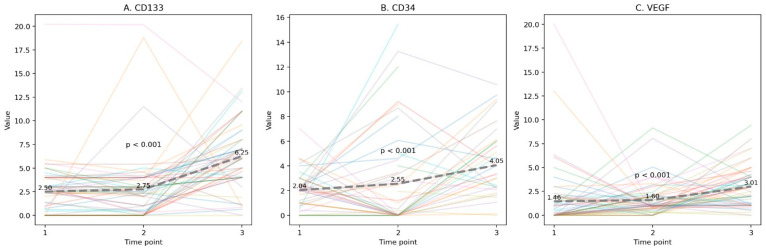
Individual patient trajectories of circulating EPC-OCN subpopulations during follow-up after TAVI. Individual values across the three study time points are shown for (**A**) CD133+/VEGFR-2+/OCN+ cells, (**B**) CD34+/VEGFR-2+/OCN+ cells, and (**C**) VEGFR-2+/OCN+ cells. Colored lines represent individual patients, and the dashed line represents the mean value at each time point. All three EPC-OCN subpopulations demonstrated a significant increase over time (*p* < 0.001 for all comparisons).

In contrast, EPCs expressing osteocalcin (EPC-OCNs) demonstrated a statistically significant increase from baseline to the late post-procedural stage (*p* < 0.01 for all markers). Specifically, CD133+/VEGFR-2+/OCN+ cells rose from a mean of 2.50% pre-procedure to 6.25% at ~90 days, CD34+/VEGFR-2+/OCN+ cells increased from 2.04% to 4.05%, and VEGFR-2+/OCN+ cells from 1.46% to 3.01% (Figure 2, Table 1). These markers represent EPCs at various stages of differentiation, where CD133+ denotes early progenitor status, CD34+ reflects a more advanced endothelial commitment, and VEGFR-2/KDR indicates endothelial lineage. The co-expression of OCN across these subsets suggests that osteogenic activity is already present at early progenitor stages and continues through more differentiated EPC populations. Individual patient trajectories across the follow-up period are shown in Figure 3, demonstrating substantial inter-individual variability together with an overall increase in EPC-OCN levels over time across all three EPC-OCN subpopulations.

### 3.3. Post-Procedural Outcomes

No deaths, major stroke, myocardial infarction, cardiogenic shock, tamponade, device migration/embolization, major bleeding, or moderate-or-greater paravalvular leak were observed during follow-up. Transient ischemic attack and acute kidney injury each occurred in 1.7% of patients. High-degree atrioventricular block requiring permanent pacemaker implantation occurred in 12.2% of cases. Red blood cell transfusion was required in 15.7% of patients, and arrhythmias were observed in 5.2%.

## 4. Discussion

In this prospective study of patients undergoing TAVI, we observed a stable profile of EPCs over time, in contrast to a significant and progressive rise in EPC-OCNs at ~90-day follow-up. This biphasic pattern highlights distinct biological roles for these cell subsets in the post-procedural setting.

Previous research has shown that classical EPCs are primarily involved in endothelial regeneration and vascular repair [8,18]. However, McDonald et al. demonstrated that endothelial regeneration in large arteries is a biphasic process driven predominantly by local endothelial cells, not by circulating progenitor cells, even under conditions of high shear stress [19]. Similarly, a study of gender-mismatched bone marrow transplant recipients found that proliferative EPCs originate from the recipient’s own vasculature, not from the bone marrow, challenging the traditional assumption regarding hematopoietic EPC origins [20]. Our finding that classical EPC levels remained unchanged after TAVI is consistent with these observations, suggesting that the procedure does not elicit a systemic mobilization of bone marrow derived EPCs, and that vascular repair may instead rely on local mechanisms.

In contrast, the marked increase in EPC-OCNs post-TAVI may reflect an osteogenic response to valve injury and remodeling. These cells co-express endothelial markers and osteogenic markers such as osteocalcin, and have been implicated in vascular calcification [10]. In an interesting study by Al-Hijji et al., EPC-OCNs were shown to increase in circulation in correlation with the severity of AS and were also identified within calcified valve tissues [12]. This supports the hypothesis that EPC-OCNs are not merely markers of disease but active participants in the pathogenesis of valvular calcification.

Further evidence supports the notion that EPC-OCNs represent a distinct subpopulation with potential pathogenic roles in cardiovascular disease. For instance, Ozcan et al. demonstrated that higher proportions of EPC-OCNs were significantly associated with increased cardiovascular risk, independent of traditional EPC levels, indicating that these cells may reflect maladaptive vascular remodeling processes [21]. These findings suggest that EPC-OCNs may not merely serve as biomarkers but could actively contribute to pathological calcification or adverse vascular outcomes in high-risk populations. This reinforces the relevance of our observation that EPC-OCNs increase significantly following TAVI, pointing to a possible mechanistic link between valve implantation and activation of osteogenic pathways.

The biological mechanisms underlying the increase in EPC-OCNs after TAVI remain incompletely understood. In the absence of functional or tissue-level validation, our findings should be interpreted as observational and hypothesis-generating rather than mechanistic. However, it is plausible that the mechanical trauma of valve implantation, changes in local hemodynamics, and possibly also inflammatory stimuli contribute to the mobilization or differentiation of osteogenic EPCs. Chan et al. proposed that EPC-OCNs possess both angiogenic and osteogenic potential and are upregulated in response to vascular injury, a theory that aligns with our observation of delayed and sustained EPC-OCN elevation post-procedure [22]. At present, EPC-OCNs are primarily assessed by flow cytometry in peripheral blood, and no established in vivo molecular imaging method is available to map their distribution in the human body. Accordingly, although our findings demonstrate dynamic changes in circulating EPC-OCN levels after TAVI, they do not provide information regarding tissue localization or homing of these cells [23]. Moreover, no tissue-level validation was available in the present study, and therefore we could not determine whether these circulating cells were present within valvular or vascular tissue after the procedure. Magnetic particle imaging has emerged as a highly sensitive preclinical approach for tracking SPION-labeled cells in vivo and may offer quantitative advantages over other imaging modalities. Experimental studies have also shown that magnetically labeled EPCs can be localized and monitored in vascular injury models. However, this technique remains investigational and has not been established for in vivo tracking of EPC-OCNs in humans [24,25]. Although EPC-based cell therapies have shown regenerative potential in ischemic cardiovascular conditions, current evidence does not support EPC-OCN transplantation as a treatment strategy in AS or after TAVI. Given the osteogenic phenotype of EPC-OCNs and their reported association with vascular and valvular calcification, any therapeutic application of this specific cell subset remains speculative and requires substantial preclinical investigation before clinical consideration [26,27].

Future studies should examine whether temporal changes in EPC-OCN subpopulations after TAVI correlate with prosthetic valve hemodynamics, residual or progressive valvular calcification, vascular complications, and longer-term cardiovascular outcomes. If validated in larger longitudinal cohorts, these circulating cell populations may have potential relevance as biomarkers of post-procedural biological remodeling rather than immediate therapeutic targets. Such an approach may help refine risk stratification and improve understanding of the biological response to valve intervention.

In summary, our findings provide new insights into the differential behavior of EPC subpopulations following TAVI. The observed stability of classical EPCs suggests a limited role for these cells in systemic vascular repair post-procedure, possibly reflecting a shift toward local tissue-driven mechanisms. The delayed but significant increase in EPC-OCNs supports the possibility that these cells may be involved in osteogenic remodeling after TAVI, although the biological and clinical significance of this finding remains uncertain. In the absence of correlations with clinical outcomes, imaging findings, or longitudinal hemodynamic parameters, the translational relevance of these observed cellular changes remains uncertain. Larger, controlled, and mechanistic studies incorporating clinical outcomes, imaging correlates, and longitudinal hemodynamic assessment are needed to better define their role in the post-TAVI setting.

## 5. Limitations

This study has several important limitations. First, the relatively small sample size and incomplete follow-up reduced the statistical power of the analysis, limited our ability to detect modest associations, and precluded more robust adjustment for potential confounders. In addition, loss to follow-up may have introduced attrition bias. Second, the single-arm observational design without a control group limits causal inference and makes it difficult to determine whether the observed changes in circulating EPC-OCN levels were specifically related to the TAVI procedure rather than to other clinical or biological factors. Third, the study did not include functional experiments or tissue-level validation. In particular, no histological analysis of valve tissue or other tissue-based assessment was available to confirm the localization or biological role of EPC-OCNs in the post-TAVI setting. Fourth, we did not evaluate the relationship between EPC-OCN dynamics and clinical outcomes, imaging findings, or longitudinal hemodynamic parameters, which limits the ability to define the clinical significance of these observations. Finally, variability in the timing of follow-up blood sampling may have influenced the measured cellular dynamics. Accordingly, our findings should be interpreted as exploratory and hypothesis-generating, and larger controlled studies with standardized longitudinal sampling and mechanistic validation are needed to confirm these observations.

## 6. Conclusions

This prospective study shows divergent temporal patterns in endothelial progenitor cell subpopulations following TAVI. While circulating EPC levels remained stable throughout follow-up, EPC-OCN levels increased significantly over time. These findings support the possibility that EPC-OCNs may be involved in post-procedural osteogenic remodeling, although the biological and clinical significance of this observation remains uncertain. Further studies are needed to clarify the mechanisms underlying EPC-OCN mobilization and to determine their potential relevance as biomarkers of biological response following TAVI.

## 7. Perspectives

Competency in Medical Knowledge: In patients undergoing TAVI, EPC-OCN levels increased over time, whereas classical EPC levels remained stable, suggesting distinct biological responses among endothelial progenitor cell subpopulations after valve intervention.

Translational Outlook: Larger studies are needed to determine whether EPC-OCN dynamics are associated with post-procedural remodeling and clinical outcomes after TAVI.

## Figures and Tables

**Table 1:** Proportion of EPC and EPC-OCN Subtypes during follow-up time period. Results represent the proportion of cells expressing the indicated markers.

	Test	Pre-Procedural			Early Post Procedural			Late Post-Procedural			*p*. Value
		Mean	N	S/D	Mean	N	S/D	Mean	N	S/D	
EPCs	34+/VEGFR-2	0.443	58	0.436	0.371	48	0.308	0.378	37	0.269	NS
EPCs	133+/VEGFR-2	0.421	58	0.351	0.333	48	0.287	0.326	37	0.214	
OCN	OCN	0.441	58	0.279	0.400	48	0.138	0.382	37	0.192	
EPC-OCN	CD133+VEGFR-2 +OCN	1.493	57	7.105	1.95	47	5.809	5.409	37	10.111	<0.01
EPC-OCN	CD34+VEGFR-2 +OCN	1.669	57	4.376	4.146	47	14.322	3.517	37	5.086	

EPC = Endothelial progenitor cell; OCN = Osteocalcin; VEGFR = Vascular endothelial growth factor; NS = Non-Significant

## Data Availability

The data presented in this study are available on request from the corresponding author due to privacy and ethical restrictions related to patient data.
